# Murine double minute 2 predicts response of advanced esophageal squamous cell carcinoma to definitive chemoradiotherapy

**DOI:** 10.1186/s12885-015-1222-0

**Published:** 2015-03-31

**Authors:** Hiroshi Okamoto, Fumiyoshi Fujishima, Takashi Kamei, Yasuhiro Nakamura, Yohei Ozawa, Go Miyata, Toru Nakano, Kazunori Katsura, Shigeo Abe, Yusuke Taniyama, Tadashi Sakurai, Jin Teshima, Makoto Hikage, Hironobu Sasano, Noriaki Ohuchi

**Affiliations:** 1 Department of Advanced Surgical Science and Technology, Graduate School of Medicine, Tohoku University, 1-1 Seiryo-machi, Aoba-ku, Sendai, 980-8574 Japan; 2 Department of Pathology, Tohoku University Hospital, Sendai, Japan; 3 Department of Pathology, Graduate School of Medicine, Tohoku University, Sendai, Japan

**Keywords:** Esophagus, Squamous cell carcinoma, MDM2, p53, p16, Ki-67, Chemoradiotherapy, Chemoradioresistance, Chemoradiosensitivity

## Abstract

**Background:**

Definitive chemoradiotherapy (dCRT) has recently become one of the most effective therapies for the treatment of esophageal squamous cell carcinoma (ESCC). However, it is also true this treatment has not been effective in all patients. Therefore, it is very important to evaluate the surrogate marker of dCRT in order to improve clinical outcomes of patients with ESCC. On the other hand, our previous study had suggested that murine double minute 2 (MDM2) and p16 were associated with chemoradioresistance in ESCC.

**Methods:**

We selected pretreatment biopsy specimens of ESCC patients from our prospective clinical study on dCRT. Seventy-nine cases histologically diagnosed as ESCC were used. We immunohistochemically investigated these specimens using antibodies against MDM2, p53, p16, and Ki-67.

**Results:**

The patients included 68 males and 11 females with a mean age of 63.3 years. The number of patients in each clinical stage was as follows: 22 in c-Stage I; 17 in c-Stage II; and 40 in c-Stage III. cT, cN, and cStage were significantly more advanced in the Failure group (including patients with persistent and recurrent disease after dCRT) than in the complete response (CR) group (patients with persistent CR after dCRT). The clinical stage inversely correlated with the CR rate and the rescue rate after failure. The overall survival rate was significantly worse in the patients with advanced cT, cN, and cStage levels, and in the Failure group. MDM2 positivity was significantly higher in the Failure group than in the CR group in cStageIII (*P* = 0.014). The number of patients with an absence of p16 immunoreactivity was significantly higher in the Failure group than in the CR group in cStageIII (*P* = 0.010) but not in cStageI or cStageII. Moreover, the overall survival with a Ki-67 ≥ 33.7% was significantly better than that with <33.7% for patients in cStageIII (*P* = 0.024).

**Conclusions:**

The results of this study suggested that MDM2 and p16 are predictive markers for chemoradioresistance in cStageIII ESCC and Ki-67 is a prognostic marker following dCRT in cStageIII ESCC. These issues could contribute to the formulation of treatment strategy for patients with advanced ESCC.

## Background

Definitive chemoradiotherapy (dCRT) has recently become one of the most effective therapies for esophageal squamous cell carcinoma (ESCC) [1]. The clinical outcomes with this treatment have been comparable with surgery alone [2]. We also reported that dCRT for patients with ESCC was comparable or even superior to surgery alone in terms of survival and quality of life [3]. However, treatment failures have also occurred following dCRT. Salvage surgery for these cases could be effective for selected patients, but this treatment has high morbidity and mortality [4]. Therefore, it has become important to estimate the possible response of ESCC to dCRT before treatment. On the other hand, by investigating the surgical specimens of salvage esophagectomies after dCRT, our previous study had suggested that murine double minute 2 (MDM2) and p16 are associated with chemoradioresistance in ESCC [5]. MDM2 directly interferes with the transcriptional activity of p53 and promotes p53 degradation by the addition of ubiquitin [6,7]. Overexpression of MDM2 has also been reported to be associated with development of radioresistance in several tumors [8]. p16 is a cyclin-dependent kinase inhibitor and its inactivation is related to carcinogenesis [9,10]. The purpose of this study was to explore whether MDM2 and p16 expression in the pretreatment biopsy specimens of ESCC patients could predict the response to dCRT or the survival of the patients after dCRT. We investigated this issue using immunohistochemical staining for MDM2, p53, p16, and Ki-67.

## Methods

### Patients and tissue samples

We selected the pretreatment biopsy specimens of ESCC patients from our prospective clinical study on CRT [3]. Briefly, eligible patients in the study were aged 20–80 years with previously untreated, T1–3 N0–3 M0 (the 7th edition of the Union for International Cancer Control system [11]), and histologically confirmed ESCC of the thoracic esophagus. The pretreatment evaluations included a barium meal, an esophagogastroduodenoscopy, a neck, chest, and abdominal computed tomography (CT), and a 2-[fluorine-18] fluoro-2-deoxy-D-glucose positron emission tomography (FDG-PET) when needed. Finally, we selected the pretreatment biopsy specimens of 79 patients, who had been histologically diagnosed with ESCC, according to the criteria among 119 cases examined.

### Definitive chemoradiotherapy and salvage esophagectomy

The CRT protocol of the prospective study basically followed the protocol of the Japan Clinical Oncology Group trial 9906 [1]. This protocol consisted of the following components: (1) 2 cycles of an intravenous cisplatin infusion (40 mg/m^2^) on days 1 and 8, (2) a continuous intravenous infusion of 5-fluorouracil (400 mg/m^2^) over 24 hours on days 1–5 and 8–12 every 5 weeks, and (3) concurrent radiotherapy (60 Gy in 30 fractions over a period of 8 weeks including a 2-week remission following the administration of 30 Gy). Salvage esophagectomy for patients with persistent or recurrent disease was performed to improve outcomes as soon as persistent or recurrent disease was confirmed, if we could obtain the informed consent from the patients and the patients were fit for the surgery [3].

### Immunohistochemistry

Immunohistochemical staining was performed using the streptavidin–biotin complex method. In brief, serial 4-μm-thick sections were deparaffinized and immersed in 3.0% hydrogen peroxide in methanol for 10 min at room temperature (RT) to block the endogenous peroxidase activity. For antigen retrieval, the slides for MDM2, p16, and Ki-67 were heated using an autoclave at 121°C for 5 min in 0.01 M citrate buffer (pH 6.0). The slides for p53 were heated in a microwave at 95°C for 15 min in 0.01 M citrate buffer (pH 6.0). Then, the slides were incubated in 1% normal rabbit serum for 30 min at RT to decrease the nonspecific antibody binding. Subsequently, the slides were incubated at 4°C overnight with mouse monoclonal antibody against MDM2 (SMP14; Santa Cruz Biotechnology Inc., CA, USA; diluted 1/1000), p53 (DO-7; Nichirei Biosciences Inc.; diluted 1/100), p16 (G175-1239; BD Biosciences; diluted 1/100), and Ki-67 (MIB-1; Dako; diluted 1/300). The next day, the sections were incubated separately with biotinylated antimouse immunoglobulin (Nichirei Biosciences Inc.) as a secondary antibody and with peroxidase-labeled streptavidin (Nichirei Biosciences Inc.) for 30 min at RT. The antigen–antibody complexes were visualized with 3,3′-diaminobenzidine.

The percentage of MDM2-, p53-, and Ki-67-positive nuclei, and p16-positive nuclei and/or cytoplasm of tumor cells was evaluated by × 400 magnification microscopy. When determining the cut-off values, we identified the values for abnormal expression as follows: p53 ≥ 10% [12] and p16 < 5% [13]. The evaluation was performed by two of the authors (HO and FF) who were blinded to the relevant clinical information of the patients examined in this study.

### Statistical analyses

All statistical analyses were performed using JMP Pro Version 9.0.2 (SAS Institute Inc., Cary, NC, USA). Continuous data were analyzed using Student’s *t*-test or the Mann–Whitney *U*-test. Categorical data were evaluated using Pearson’s chi-square test, Fisher’s exact test, or the Mann–Whitney *U*-test as appropriate. Normality was assessed using the Shapiro–Wilk test. Equality of variances was evaluated using the F test. Overall survival (OS) curves were determined by the Kaplan–Meier method, and a log-rank test was used to compare the survival curves. A *P* value of <0.05 was considered statistically significant. The patient survival time was determined from the date of the start of treatment until death or the last follow-up examination.

This study was approved by the Ethical Committee of Tohoku University Hospital (accession number 2011–596).

## Results

### Patient characteristics and clinical courses

**Figure 1:**
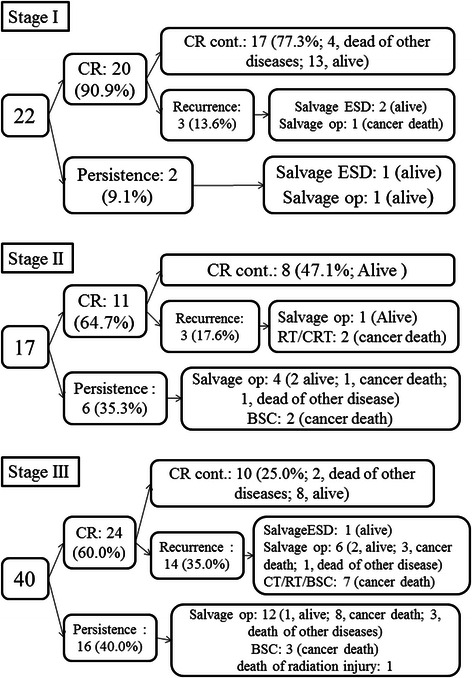
**Clinical course of the patients according to their clinical stage.** CR, complete response; CR cont., Complete response continued; ESD, endoscopic submucosal dissection; op, operation; CT, chemotherapy; RT, radiotherapy; CRT, chemoradiotherapy; BSC, best supportive care.

Table 1 summarizes the clinicopathological findings of the patients examined in this study. The patients included 68 males and 11 females with a mean age of 63.3 years (range, 43–79 years). The number of patients in each clinical stage was as follows: 22 in c-Stage I; 17 in c-Stage II (7, T3N0; 6, T1N1; 4, T2N1); and 40 in c-Stage III (35, T3N1; 5, T3N2). The CR (complete response) group contained the patients who were determined to have a CR after dCRT and in whom CR had been continued. Failure group included the patients with persistent and recurrent disease after dCRT. The clinical courses of the patients according to clinical stage are shown in Figure 1. The clinical stage inversely correlated with the CR rate and the rescue rate following the therapeutic failure. Table 1 also shows that the patients with more advanced cT, cN, and cStage levels had more possibility to lead to Failure group rather than CR group. Moreover, such patients had a significantly worse prognosis compared with CR group (Table 2).

**Table 1:** **Characteristics of the CR group and failure group**

Variables	Total (n = 79) (%)	CR group (n = 35) (%)	Failure group (n = 44) (%)	*P*value*
Age (years)				
mean ± SD	63.3 ± 8.3	63.6 ± 8.8	63.0 ± 7.9	0.73
(Range)	(43–79)	(48–79)	(43–79)	
Gender				
Male	68 (86.1)	30 (85.7)	38 (86.4)	1.00
Female	11 (13.9)	5 (14.3)	6 (13.6)	
Location				
Upper	12 (15.2)	5 (14.3)	7 (15.9)	0.98
Middle	47 (59.5)	21 (60.0)	26 (59.1)	
Lower	20 (25.3)	9 (25.7)	11 (25.0)	
cT				
cT1	24 (30.4)	19 (54.3)	5 (11.4)	<0.0001
cT2	8 (10.1)	4 (11.4)	4 (9.1)	
cT3	47 (59.5)	12 (34.3)	35 (22.7)	
cN				
cN0	29 (36.7)	19 (54.3)	10 (22.7)	0.0037
cN1-3	50 (63.3)	16 (45.7)	34 (77.3)	
cStage				
cStage I	22 (27.8)	17 (48.6)	5 (11.4)	0.0004
cStage II	17 (21.5)	8 (22.9)	9 (20.5)	
cStage III	40 (50.6)	10 (28.6)	30 (68.2)	

CR, complete response; SD, standard deviation.

**Table 2:** **Overall survival with clinicopathological findings**

Variables	n = 79 (%)	3-years OS rate (%)	5-year OS rate (%)	*P*value*	
Age (years)					
<60	27 (34.2)	70.4	63.0	0.11	
≥60	52 (65.8)	52.5	43.1		
Gender					
Male	68 (86.1)	58.9	50.8	0.91	
Female	11 (13.9)	57.7	46.2		
Location					
Upper	12 (15.2)	50.0	41.7	control	
Middle	47 (59.5)	61.0	48.2	0.76	
Lower	20 (25.3)	60.0	60.0	0.38	
cT					
cT1	24 (30.4)	85.9	76.4	control	
cT2	8 (10.1)	85.7	71.4	0.94	
cT3	47 (59.5)	41.5	34.4	0.0006	
cN					
cN0	29 (36.7)	80.8	72.7	0.0028	
cN1-3	50 (63.3)	47.0	38.2		
cStage					
cStageI	22 (27.8)	89.5	79.0	control	
cStageII	17 (21.5)	67.0	60.3	0.25	
cStageIII	40 (50.6)	40.0	31.8	0.0002	
Clinical course					
CR continue	35 (44.3)	87.5	84.1	<0.0001	
Failure	44 (55.7)	37.3	25.5		

*log-rank test.

OS, overall survival.

### Comparison of marker expression between the CR group and failure group

**Figure 2:**
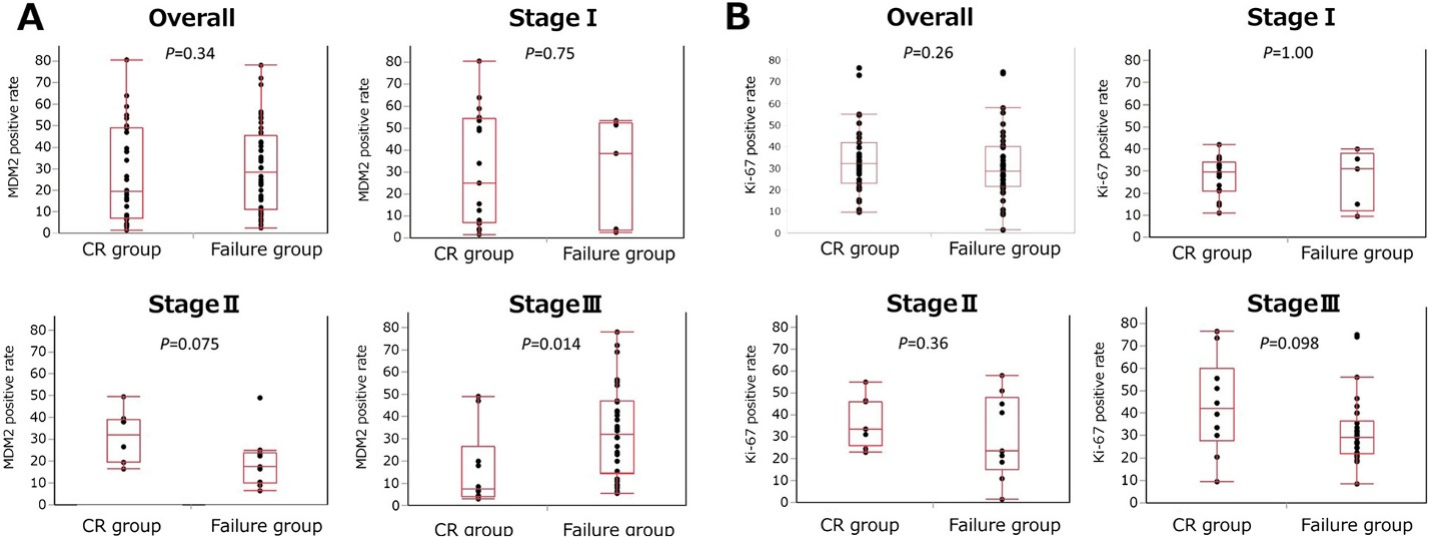
**Comparison of marker expression between CR group and Failure group.** The MDM2-positive rate of the Failure group was significantly higher than that of the CR group in cStageIII disease (*P =* 0.014). This was not seen in the overall group and in cStageI and cStageII cases **(A)**. The Ki-67-positive rate of the CR group tended to be higher than that of the Failure group in cStageIII cases (*P =* 0.098), but it did not reach statistical significance. This was not seen in the overall cohort and patients with cStageI and cStageII **(B)**.

**Figure 3:**
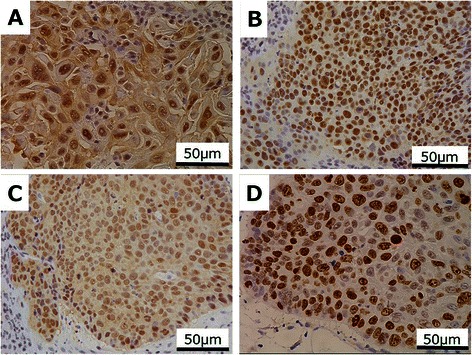
**Immunohistochemical staining of esophageal squamous cell carcinoma.** Tumor cells positive for MDM2 **(A)**, p53 **(B)**, p16 **(C)**, and Ki-67 **(D)** expression (×400 magnification).

The MDM2-positive rate in the Failure group was significantly higher than that in CR group in cStageIII (*P =* 0.014). This was not the case in the entire group, or in those patients with cStageI or cStageII disease (Figure 2A). The p53 expression did not demonstrate any correlations between the CR group and the Failure group (Table 3). The number of patients that were p16 negative was significantly higher in the Failure group than in the CR group in the patients with cStageIII disease (*P =* 0.010). This finding was not seen in the complete group or in patients with cStageI and cStageII disease (Table 3). The Ki-67-positive rate of the CR group tended to be higher than that of the Failure group in patients with cStageIII disease (*P =* 0.098); however, this did not reach statistical significance. This tendency was not seen in the overall group or in patients with cStageI or cStageII disease (Figure 2B). Representative illustrations of immunohistochemistry are presented in Figure 3.

**Table 3:** **Comparison of p53 and 16 expression between the CR group and failure group**

Variables		CR group	Failure group	*P*value
p53				
Overall	negative	12	15	0.99
	positive	23	29	
StageI	negative	6	0	0.27
	positive	11	5	
StageII	negative	3	2	0.62
	positive	5	7	
StageIII	negative	3	13	0.71
	positive	7	17	
p16				
Overall	negative	29	39	0.52
	positive	6	5	
StageI	negative	16	5	1.00
	positive	1	0	
StageII	negative	7	5	0.29
	positive	1	4	
StageIII	negative	6	29	0.01
	positive	4	1	

CR, complete response.

**Figure 4:**
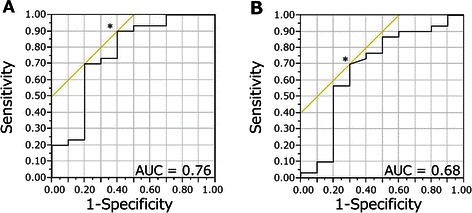
**ROC curve of MDM2 and Ki-67 in cStageIII.** The receiver operating characteristic (ROC) curve analysis of MDM2 positivity in cStageIII patients identified a cut-off for the Failure group at 9.05% that provided 90% sensitivity and 60% specificity, with a positive predictive value (PPV) of 87.1% and a negative predictive value (NPV) of 66.7%. The area under the curve (AUC) was 0.76 **(A)**. The ROC curve analysis of Ki-67 positivity in cStageIII identified a cut-off for the CR group at 33.7% that provided 70% sensitivity and 70% specificity, with a PPV of 43.8% and a NPV of 87.5%. The AUC was 0.68 **(B)**.

In terms of MDM2 expression in cStageIII, the receiver operating characteristic (ROC) curve analysis identified a cut-off for the Failure group at 9.05% that provided 90% sensitivity and 60% specificity, with a positive predictive value (PPV) of 87.1% and a negative predictive value (NPV) of 66.7%. The area under the curve (AUC) was 0.76 (Figure 4A). For Ki-67 expression in cStageIII, the ROC curve analysis identified a cut-off for the CR group of 33.7% that provided 70% sensitivity and 70% specificity, with a PPV of 43.8% and a NPV of 87.5%. The AUC was 0.68 (Figure 4B).

### Correlation between marker expressions

**Figure 5:**
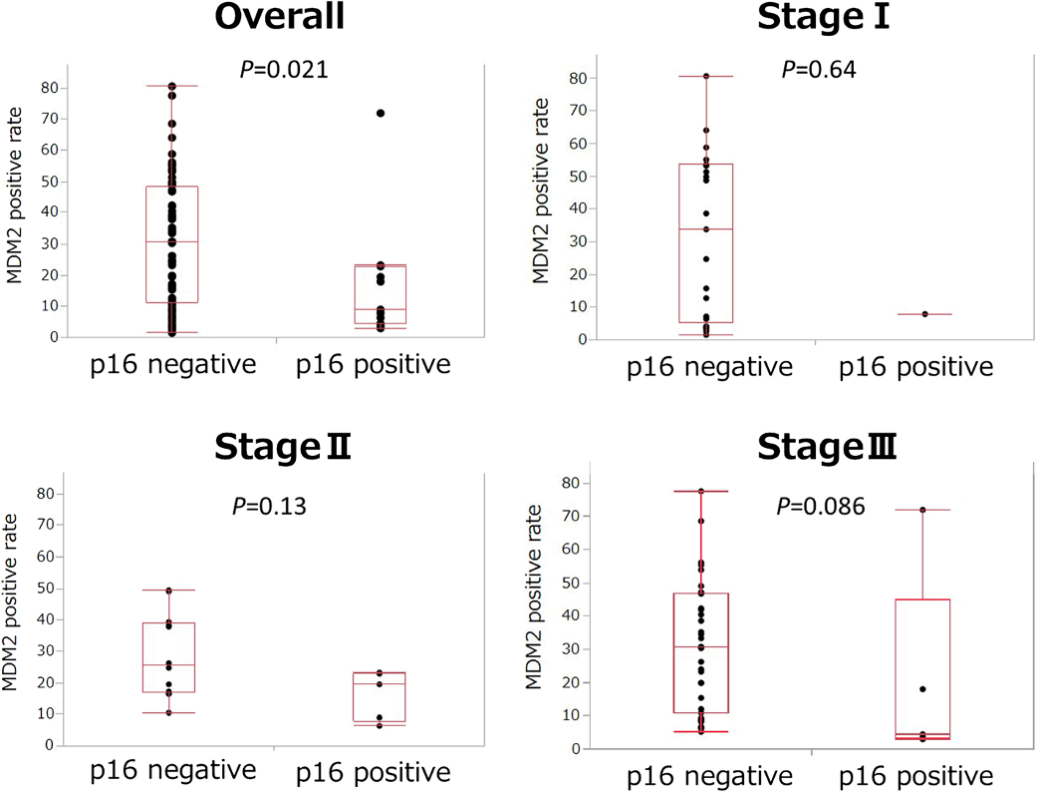
**Correlation between MDM2 and p16.** MDM2-positive rate tended to be higher in the p16-negative group than in the p16-positive group in the overall group (*P =* 0.021) and in cStageIII patients (*P =* 0.086). This was not seen in cStageI and cStageII cases.

In terms of correlation between marker expressions, the MDM2-positive rate tended to be higher in the p16-negative group than in the p16-positive group in the overall group of patients (*P =* 0.021) and the cStageIII patients (*P =* 0.086) (Figure 5). This was not the case for cStageI and cStageII patients. No significant correlations were found among the other marker expressions with either the overall group or each cStage group (data not shown).

### Survival analysis to marker expression

**Figure 6:**
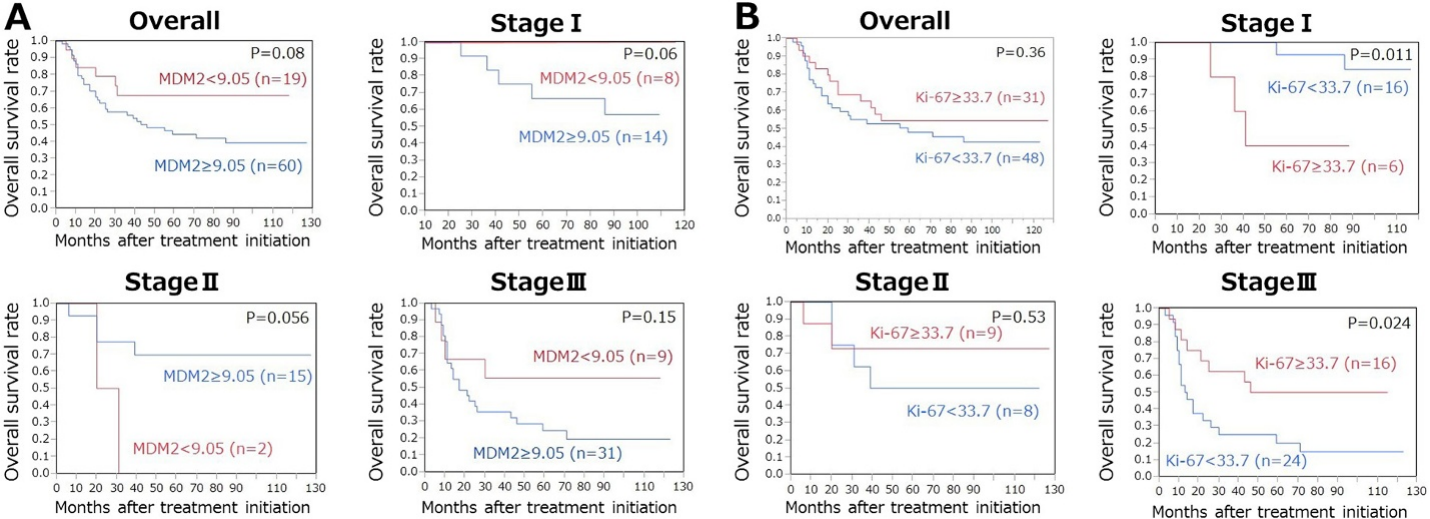
**Kaplan–Meier curves of patients prepared on the basis of MDM2 and Ki-67.** Overall survival of the patients with MDM2 levels ≥9.05% was tended to be worse than that of the patients with levels <9.05% in the overall group (*P* = 0.08), and in cStageI (*P* = 0.06) and cStageIII cases (*P* = 0.15), but that did not reach statistical significance **(A)**; the overall survival of the patients with Ki-67 levels ≥33.7% was significantly better than that of patients with levels <33.7% in cStageIII cases (*P* = 0.024), but the finding was opposite for cStageI patients (*P* = 0.011) **(B)**.

In terms of MDM2 positivity, the OS of the patients with MDM2 levels ≥9.05% was worse than that of the patients with levels <9.05% in the overall cohort (*P* = 0.08), and patients in cStageI (*P* = 0.06), and cStageIII (*P* = 0.15); however, these numbers did not reach statistical significance (Figure 6A). The two patients with MDM2 levels <9.05% in the cStageII group consisted of a patient who had rejected salvage treatment for persistent disease and a patient who had died of another disease after a short period. In terms of Ki-67, the OS of the patients with Ki-67 levels ≥33.7% was significantly better than that of patients with levels <33.7% in the cStageIII group (*P* = 0.024); however, the findings were opposite in cStageI disease (*P* = 0.011) (Figure 6B). The patients with Ki67 levels ≥33.7% in the cStageI group included a patient who underwent salvage esophagectomy for recurrent disease and died of cancer, and two patients who had continued CR but died of other diseases.

## Discussion

The clinical course in this study showed that in earlier stages of tumor progression, the dCRT response rate was higher; moreover, the lifesaving rate of salvage treatment for the persistent and recurrent cases was also higher. In contrast, the response rate of the patients with advanced cancer, such as those with cStageIII disease, was markedly reduced and the prognosis of the patients with persistent and recurrent disease was also poorer. In addition, as the cT, cN, and cStage levels advanced, the prognosis became worse; the prognosis of the CR group was significantly better than that of the Failure group. These results revealed that the chemoradioresistance of ESCC may partially depend on the status of tumor progression. According to one theory, the radiosensitivity of breast cancer depends on specific factors including tumor size [14]. It would be clinically useful if we could predict the chemoradiosensitivity of ESCC using a scoring method of clinical and pathological factors such as the Van Nuys Prognostic Index in breast cancer [15]. The correlation between MDM2 expression and the prognosis of ESCC has been reportedly controversial [16-18]. However, our study suggested that MDM2 expression could be a potent predictive marker for chemoradioresistance for advanced ESCC. We may be able to consider treatment strategy while remembering that it is highly probable that cStageIII cases with high MDM2 positivity will show chemoradioresistance. Using this strategy may suggest surgery-based treatment with/without neoadjuvant or adjuvant therapy for these cases. Although patients with high MDM2 positivity may obtain a CR state clinically, it is still necessary to closely observe them because these patients may be at high risk for cancer recurrence. The salvage treatment should be performed immediately after any clinical signs suggestive of recurrence appear, which could contribute to the improvement of the survival rate. A prospective study will be needed to evaluate these findings. Recently, MDM2 inhibitor Nutlin-3 has attracted some attention in the field of leukemia as an anti-tumor agent [19]. In addition, it has also been reported that Nutlin-3 improved the radiosensitivity of laryngeal squamous cell carcinoma, which shares many biological characteristics with ESCC [20]. It is certainly considered clinically worthwhile to attempt the clinical study of this inhibitor in patients with ESCC.

For p16, a low CRT effect was observed in p16-negative tumors only in cStageIII cases, similar to MDM2. Although there were issues that will need to be addressed such as the high number of patients that were p16-negative and the small number of patients in this study, we found that p16 could also be a predictive marker for chemoradiosensitivity in advanced ESCC. No correlation was observed between chemoradiosensitivity and the status of both MDM2 and p16 in patients with cStageI and cStageII disease. Generally, the evaluation of immunostaining for dysplasia and carcinoma *in situ* is often difficult. MDM2 was highly expressed in so-called squamous dysplasia and carcinoma *in situ* [21,22]. MDM2 overexpression could play different roles in early tumorigenesis and development of chemoradioresistance or sensitivity in ESCC. Further research will be needed for the evaluation of MDM2 overexpression and its relevance to chemoradioresistance. From this point of view, the biopsy specimen should be taken from the invasive section of the tumor in advanced ESCC to completely evaluate this immunostaining.

Cut-off values between 10% and 50% for MDM2 with respect to carcinogenesis, prognosis, and chemoradioresistance have been used [17,23-25]. We attempted to calculate the cut-off value using the ROC curve. It is conceivable that approximately 10% may be an appropriate cut-off value for prognosis and chemoradiosensitivity. Previous research has reported that high Ki-67 levels were correlated with a good response to CRT [26-28]. Imdahl et al. reported that the cut-off value of Ki-67 for responsiveness of esophageal cancer to neoadjuvant chemoradiotherapy was 39% [27]. The cut-off value from this study (33.7%) that was calculated using the ROC curve was also consistent with that reported by Imdahl et al. The results of the previous study for prognosis and chemoradiosensitivity followed the same trend as our current study.

Looking at the association between markers, there was no correlation between MDM2 and p53 expression in this study. The positive correlation of MDM2 and p53, and the p53-dependent and -independent role of MDM2 in ESCC has been previously reported [29,30]. Because the number of cases evaluated in this study was rather limited, further investigation will be required for these issues. On the other hand, a relationship between MDM2 and p16 has been suggested by this study. Besides inhibiting p53, MDM2 can also inhibit the cell cycle in another route; for example, MDM2 directly inhibits the retinoblastoma protein [31,32]. It has also been reported that both p16 and p14 (ARF), which inactivate MDM2, are on the same chromosome 9p21 [33]. However, further examination on the relationship between these peripheral markers is required.

## Conclusions

In conclusion, the results of this study suggest that MDM2 and p16 might have potential as predictive markers for chemoradioresistance in cStageIII ESCC and also that Ki-67 may also have a role as a putative prognostic marker following dCRT in cStageIII ESCC. Future studies might incorporate these potential biomarkers in trials to determine their effectiveness in formulating treatment strategies for patients with advanced ESCC. We hope that this study contributes to the treatment strategy of ESCC.

## Abbreviations

dCRT: Definitive chemoradiotherapy
ESCC: Esophageal squamous cell carcinoma
MDM2: Murine double minute 2
RT: Room temperature
OS: Overall survival
CR: Complete response
ROC: Receiver operating characteristic
PPV: Positive predictive value
NPV: Negative predictive value
AUC: Area under the curve.
